# Supplementary figures and images for: Ananke: temporal clustering reveals ecological dynamics of microbial communities
Source: PeerJ. 2017 Sep 26;5:e3812. doi: 10.7717/peerj.3812 (PMC5621509; doi:10.7717/peerj.3812)

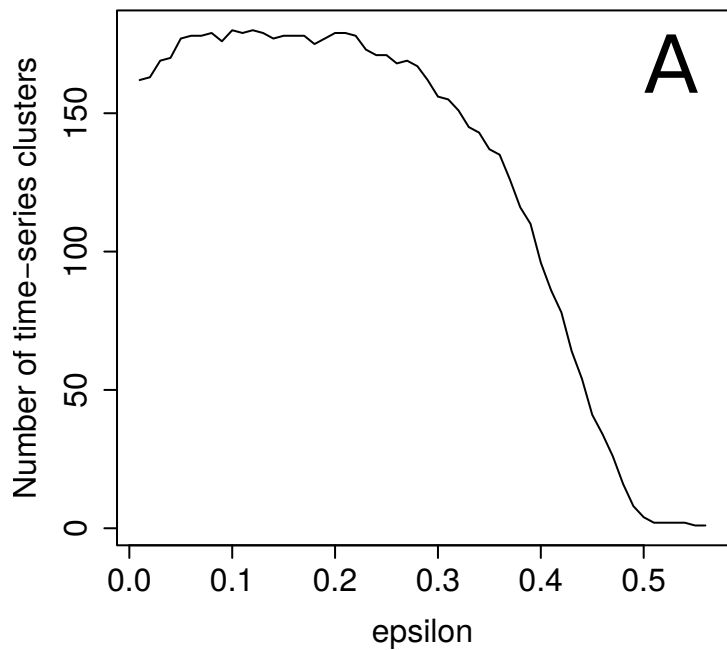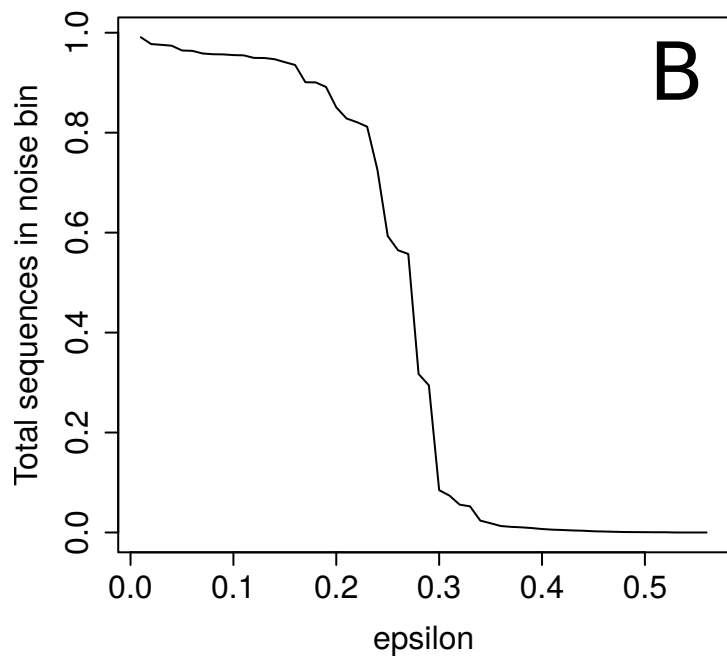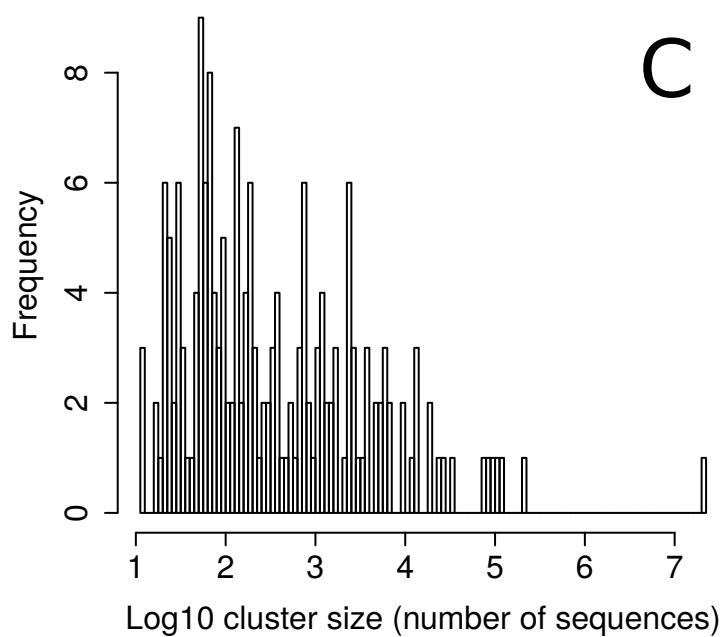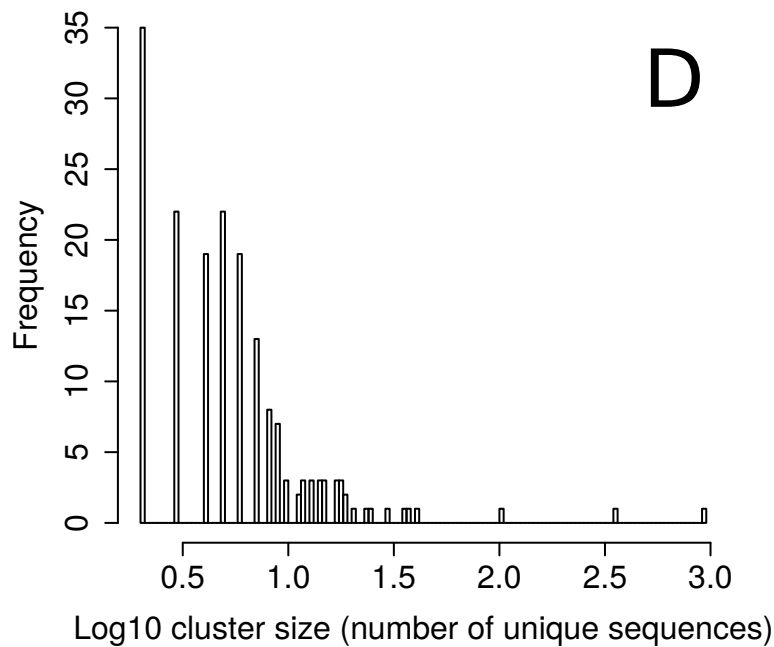

Supplement: Figure S1 — Time-series cluster descriptions for the faecal sample data, pre-processed with DADA2 denoising. (A) Number of time-series clusters as a function of the clustering parameter, ε. (B) Proportion of sequences in the “noise bin” as a function of the clustering parameter, ε. (C) Distribution of the sizes of time-series clusters (in log10 number of total sequences). (D) Distribution of the sizes of time-series clusters (in log10 number of unique sequences). [file peerj-05-3812-s001.pdf]

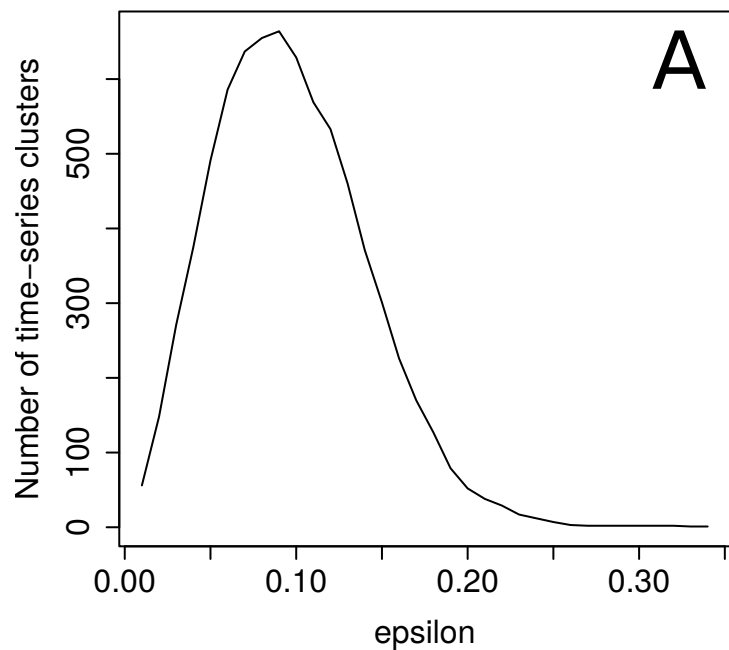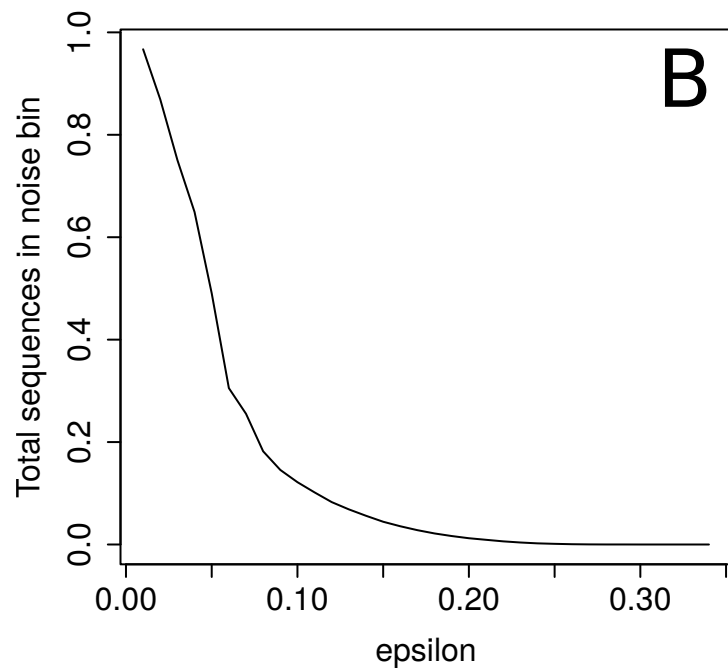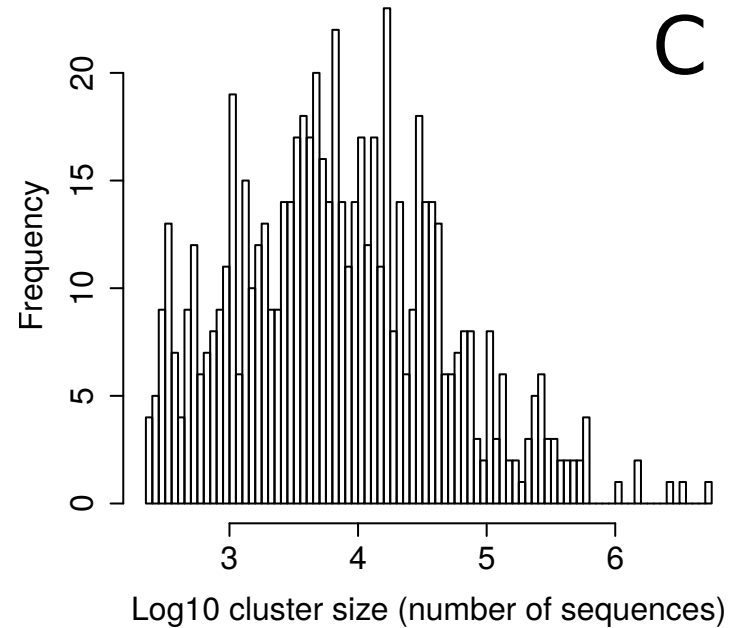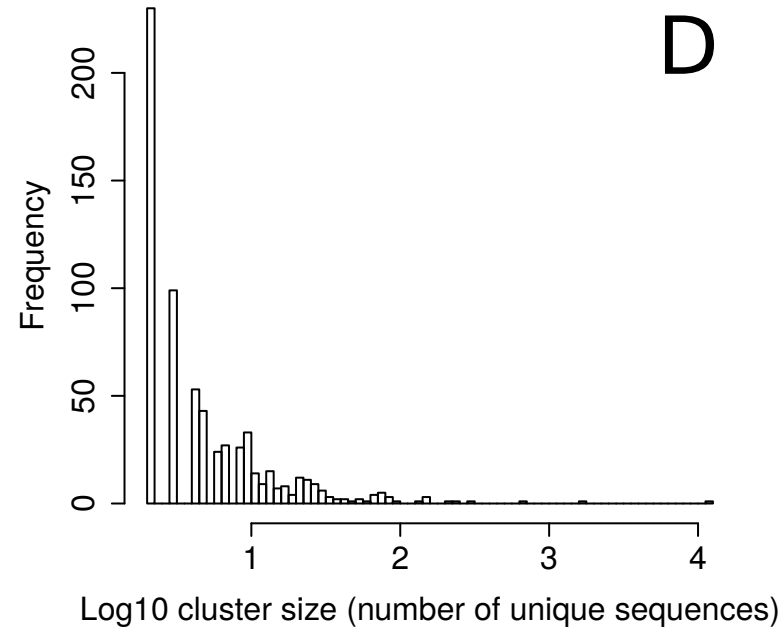

Supplement: Figure S2 — Time-series cluster descriptions for the freshwater lake data. (A) Number of time-series clusters as a function of the clustering parameter, ε. (B) Proportion of sequences in the “noise bin” as a function of the clustering parameter, ε . (C) Distribution of the sizes of time-series clusters (in log10 number of total sequences). (D) Distribution of the sizes of time-series clusters (in log10 number of unique sequences). [file peerj-05-3812-s002.pdf]
